# Lessons from the pandemic on the value of research infrastructure

**DOI:** 10.1186/s12961-021-00704-2

**Published:** 2021-04-01

**Authors:** Laurence S. J. Roope, Paolo Candio, Vasiliki Kiparoglou, Helen McShane, Raymond Duch, Philip M. Clarke

**Affiliations:** 1 Health Economics Research Centre, Nuffield Department of Population Health, University of Oxford, Old Road Campus, Headington, Oxford, OX3 7LF UK; 2 National Institute for Health Research Oxford Biomedical Research Centre–John Radcliffe Hospital, Oxford, UK; 3 Nuffield Department of Primary Health Care Sciences, University of Oxford, Oxford, UK; 4 Nuffield Department of Medicine, University of Oxford, Oxford, UK; 5 Nuffield College, University of Oxford, Oxford, UK; 6 Centre for Health Policy, Melbourne School of Population and Global Health, University of Melbourne, Melbourne, VIC Australia

**Keywords:** Option value, Research funding, Research infrastructure, Resilience

## Abstract

The COVID-19 pandemic has shed a spotlight on the resilience of healthcare systems, and their ability to cope efficiently and effectively with unexpected crises. If we are to learn one economic lesson from the pandemic, arguably it is the perils of an overfocus on short-term allocative efficiency at the price of lack of capacity to deal with uncertain future challenges. In normal times, building spare capacity with ‘option value’ into health systems may seem inefficient, the costs potentially exceeding the benefits. Yet the fatal weakness of not doing so is that this can leave health systems highly constrained when dealing with unexpected, but ultimately inevitable, shocks—such as the COVID-19 pandemic. In this article, we argue that the pandemic has highlighted the potentially enormous option value of biomedical research infrastructure. We illustrate this with reference to COVID-19 response work supported by the United Kingdom National Institute for Health Research Oxford Biomedical Research Centre. As the world deals with the fallout from the most serious economic crisis since the Great Depression, pressure will soon come to review government expenditure, including research funding. Developing a framework to fully account for option value, and understanding the public appetite to pay for it, should allow us to be better prepared for the next emerging problem.

## Main text

The coronavirus disease 2019 (COVID-19) pandemic has shed a spotlight on the resilience of healthcare systems, and their ability to cope efficiently and effectively with unexpected crises. If we are to learn one economic lesson from the pandemic, arguably it is the perils of an overfocus on short-term allocative efficiency at the price of lack of capacity to deal with uncertain future challenges.

In a seminal study, Weisbrod developed the concept that has evolved into ‘option value’ [1]. The insight is that there can be value in having access to use of a public good or service, even if there is uncertainty as to whether or not it will ever actually be used. For example, rather like insurance, investing in spare capacity for emergency services may seem inefficient in the short term, but has clear option value due to the ability it provides to respond to uncertain emergencies.

The concept of option value has evolved into two broad separate, though closely related, versions. One version, sometimes referred to as ‘quasi-option value’, emerged mainly from the literature on environmental economics and has emphasized the so-called ‘irreversibility effect’ of environmental degradation from certain investments [2–4]. A second version, sometimes referred to as ‘real option value’ was motivated mainly by business investment decisions, where the future value of an irreversible investment is uncertain [5]. This version developed the similarity between investment decisions and financial options. The two concepts are very similar, with only subtle differences. Quasi-option value can be characterized as capturing the value of learning (and so reducing uncertainty) while preserving options; real option value captures the value of preserving options, conditional on learning [6]. Common to both concepts is that irreversibility of decisions, in the sense of removing future options, together with uncertainty over the consequences of irreversibility, adds to the value of preservation [6]. In this article, in contrast to considering investment decisions that may irreversibly erode option value, we will consider the option value that investment in a public good can provide.

The pandemic has presented some obvious examples of goods with major option value. Many countries have struggled, for example, with shortages of personal protective equipment, health professionals, intensive care unit beds and mechanical ventilators—not to mention capacity to test for the virus [7]. In the case of ventilators, there is evidence that the problem was not necessarily shortage of actual numbers, but rather a lack of information infrastructure [8]. In the United Kingdom, in the first wave of the pandemic, occupancy of beds compatible with mechanical ventilation never exceeded 62% at the national level, yet 30% of hospitals across England reached full saturation at some point [8]. Investing in infrastructure that makes real-time bed occupancy data available to front-line workers could have enabled nearby hospitals to ease pressure on those exceeding recommended bed occupancy. In normal times, building capacity in such ways may seem inefficient, the costs potentially exceeding the benefits. Yet the fatal weakness of not adopting such strategies is that this can leave healthcare systems highly constrained when dealing with unexpected, but ultimately inevitable, shocks—such as the COVID-19 pandemic. While in the last year there have been some extraordinary efforts to expand capacity [9–11], better preparation, including development of infrastructure that would make capacity available when needed, would have saved many lives as well as, in the long run, being more efficient economically.

We argue that a less obvious—but perhaps no less important—lesson from the pandemic on infrastructure is on the option value of research. At the University of Oxford, for example, researchers working on high-profile projects, such as the Oxford vaccine [12], the RECOVERY (Randomized Evaluation of COVID-19 Therapy) trial [13] and the Office for National Statistics (ONS) Coronavirus Infection Survey [14], have been supported by the National Institute for Health Research (NIHR) Oxford Biomedical Research Centre (Oxford BRC).

The Oxford BRC is a partnership between the University of Oxford and Oxford University Hospitals National Health Service (NHS) Foundation Trust. The overall aim of the NIHR Oxford BRC, which is ultimately funded through the United Kingdom Department of Health and Social Care, is to translate basic scientific developments and laboratory research into clinical benefits and the clinical setting. It is one of 20 BRCs in England to have received funding during 2017–2022, following a competitive bidding process. It is divided into 20 themes and four clusters: Precision Medicine; Technology and Big Data; Immunity and Infection; and Chronic Diseases. Its total funding during 2017–2022 comprises around £114 m.

In contrast to funding granted to conduct specific studies (which cannot generally be diverted), research infrastructure funding has the flexibility to facilitate researchers to respond quickly and effectively to uncertain major health issues as they arise, allowing pilot studies to be undertaken while seeking dedicated funding. In the case of COVID-19, the enabling nature of research infrastructure funding meant that some funding could be repurposed at short notice to tackle the emergency. Thus, a number of research groups were able to rapidly divert resources from existing projects to address an emerging issue. Subsequently, many received significant funds from other sources, but without the initial infrastructure support critical time would have been lost. Further, data infrastructure, such as NHS DigiTrials [15], has allowed rapid analysis by having a system that routinely links administrative data to outcomes of participants in clinical trials.

Quantifying the value of research in general [16], and research infrastructure in particular, is a difficult multidimensional and intertemporal problem that is gaining increasing attention [17, 18]. Using the Oxford BRC as an example, we posit that a major overlooked source of value of research funding in general, and research infrastructure in particular, is its option value. Specifically, the option value from Oxford BRC funding attributable to COVID-19 could be staggeringly high. Consider first the prospect of the Oxford vaccine making a substantial contribution towards ending the pandemic. This now seems likely given that it has been found effective in Phase II/III trials [19] and is substantially cheaper and more easily stored than Pfizer and Moderna’s effective mRNA vaccines [20]. The International Monetary Fund (IMF) has estimated that COVID-19 will decrease world economic output by a total US$ 11 trillion in 2020 and 2021 [21]. This amounts to US$ 458 billion a month. A simple calculation suggests that if BRC infrastructure sped up the development of an effective vaccine by just 1 day, the value to the global economy could be up to US$ 15 billion. To place this in the context of expenditure, the total Oxford BRC budget for 2017–2022 is less than 1% of this. Here we use the speeding up of the vaccine development by ‘just 1 day’ simply as an illustrative benchmark of the very large value that would arise from speeding up vaccine development by even a very small amount of time. Detailed empirical analysis would be required to give a realistic estimate of by quite how much time research infrastructure sped up the development of the Oxford vaccine. Such an estimate would be an important input to a cost–benefit analysis.

Another contribution to tackling the pandemic made by researchers supported by Oxford BRC funding is the evidence from the RECOVERY trial that dexamethasone reduces death by up to one third in hospitalized patients with severe respiratory complications of COVID-19 [13]. Again, a framework for valuing research infrastructure could encompass the degree to which it has been able to accelerate the development of studies such as the RECOVERY trial and thereby produce knowledge that can potentially save lives. While no economic evaluation has yet been conducted, dexamethasone is a drug that is both low cost and widely available and, according to the study’s chief investigator, for less than £50 (US$ 63), eight patients can be treated and one life can be saved [22].

A full assessment of the value of Oxford BRC research infrastructure would require a detailed cost–benefit analysis or other economic evaluation. This analysis would need to account for both uncertainty and reasonable counterfactuals. Identifying an appropriate counterfactual is not easy, but it seems likely that over the course of the pandemic, a delay to the Oxford vaccine would have led to increased time spent under the sorts of pandemic management approaches we have actually observed—more time in lockdown, more social distancing, more mask wearing and so on. In short, the pandemic would have been longer and the associated costs would have been greater.

We cannot rule out the possibility that a delayed Oxford vaccine would have led to greater investment in research on treatments, which might have mitigated these extra costs. However, this counterfactual scenario seems questionable. As discussed above, BRC research infrastructure has itself made a substantial contribution to research on treatment by facilitating the RECOVERY trial, which provided the first trial-based evidence of an effective treatment for COVID-19 (dexamethasone). Thus, it is quite possible that without BRC research infrastructure, there would actually have been less—not more—research on treatment.

It is also conceivable that a delayed Oxford vaccine might have led to increased research that would speed up the development of other COVID-19 vaccines—and so mitigate the costs from the delay. The likelihood of this counterfactual scenario is difficult to ascertain.

Beyond economic evaluation, another possible approach to assessing the option value of research infrastructure would be to elicit the public’s willingness to pay for it. At the time of writing, for example, as part of the CANDOUR (COVID-19 Vaccine Preference and Opinion Survey) study [23], we are in the process of collecting stated preference data from 13 countries on the willingness of the public to pay additional taxes to build resilience to the spreading of a future pandemic. At the least, knowledge of the public’s willingness to pay for such resilience could help policy-makers gauge the likely political acceptability of increased investments in infrastructure, and of the possible communication challenges that may be needed to explain their value to the public.

## Conclusions

The role of the Oxford BRC in tackling the COVID-19 pandemic emphasizes the potentially enormous option value that biomedical research infrastructure can provide. It is important to note that pandemic preparedness is just one example of the option value of research infrastructure. The world faces similar challenges with antibiotic resistance [24] and, again, needs the flexibility to scale up research to address problems either as they emerge, or in anticipation (e.g. developing infrastructure for the testing of new antibiotics).

As the world deals with the fallout from the most serious economic crisis since the Great Depression, pressure will soon come to review government expenditure, including research funding. Developing a framework to fully account for option value, and understanding the public appetite to pay for it, should allow us to be better prepared for the next emerging problem.

## Data Availability

Not applicable.

## Abbreviations

BRC: Biomedical Research Centre
CANDOUR: COVID-19 Vaccine Preference and Opinion Survey
COVID-19: Coronavirus disease 2019
IMF: International Monetary Fund
NIHR: National Institute for Health Research
NHS: National Health Service
ONS: Office for National Statistics
RECOVERY: Randomized Evaluation of COVID-19 Therapy
